# Fractures of the Vertebræ

**Published:** 1829-03

**Authors:** 


### HOSPITAL REPORTS,

*(Principally condensed from various Periodical Publications.)*

---

### FRACTURES OF THE VERTEBRÆ.

### *Fracture of the Bodies of the Seventh and Eighth Dorsal Vertebræ.*

(St. Bartholomew's Hospital.)

William Fruig, æt. thirty-seven, a labouring man, was admitted on the morning of the 6th January, under the care of Mr. Lawrence, having fallen from the top of a house upon his back, a height of about fifty feet. As he lay upon the bed, the lower extremities had very much the appearance of having been fractured, particularly the femur of the right side, but, when examined, they were found to be quite sound. The power of motion and sensation were completely gone, so much so that he was unconscious of a considerable-sized wound upon the great toe. This loss of sensation extended all over the front of the abdomen and around the loins, as high up as the scrobiculus cordis. There was, however, perfect sensation above this part. Passing the hand up the spinous processes of the vertebræ of the loins and back did not produce any sensation or pain, until it arrived opposite to about the seventh or eighth dorsal vertebræ, where there was felt a slight depression, and a considerable degree of pain was occasioned by making the slightest pressure. The pulse was slow and labouring; the respiration not much disturbed.

7th.—No return of sensation; much pain about the part of the back that is injured; has also a distressing sensation in the abdomen, above the navel. The belly here is distended, and forms a complete contrast with the lower part of the abdomen, which is flat and senseless. Much pain in the bowels. No evacuation. Urine passed insensibly. Pulse rather quick. No appetite.—Ordered house medicine every three hours.

In the evening, there was more pain about the bowels. There had been no evacuation, and the skin was rather hot.—Ordered an enema.

8th.—The bowels have been freely opened. There is still much pain above the navel, and the abdomen is generally tense, owing to the distended state of the bladder, which had not relieved itself. By pressing the hand upon the distended viscus, no urine could be forced through the passage; so that a catheter was introduced, and about three pints of offensive high-coloured urine drawn off, which afforded some relief. Pulse slow. The stools pass involuntarily.

10th.—Little alteration, except that he complains of pain in the throat and chest.

12th.—The pain in the throat has disappeared, but it is severe in the chest. Countenance anxious; has had no sleep; stools continue to pass involuntarily. The urine is drawn off twice a day.

13th.—Pain in the chest increased. Pulse ninety, and full.—V.S. ad ℥viij.

15th.—The urine may now be pressed out of the bladder by placing the hand over the lower part of the abdomen, so that the catheter is dispensed with. Pulse slow; much thirst.

From this time the only variations in his symptoms were those of his pulse becoming quicker, and his breathing more difficult. He died on the morning of the 19th, having survived the accident twelve days.

On examining the body six hours after death, the spine was found to have a lateral curvature, which did not seem to have been caused by the injury. Upon cutting the muscles on each side of the dorsal vertebræ, much blood was seen to be extravasated in the cellular tissue around them, particularly in the neighbourhood of the seventh and eighth, the spinous processes of which were quite moveable before the saw was applied. There seemed to be so much mischief done that it was not easy to discover what was the exact injury, but the oblique processes of the eighth vertebræ seemed to be driven upwards, over part of the body of the one above. The theca about this part was of a dark red appearance, and seemed to be pressed upon by the posterior part of the body of the vertebræ, so as to cause a sharp edge to be formed. There was much blood effused beneath the arachnoid, and that portion of the cord which was opposed to the sharp edge of the vertebræ was pressed upon and softened, so as to bear no resemblance to the cord above this part; cutting into it showed several bloody points dispersed over it. Removing the cord showed the fracture to extend across the bodies of the seventh and eighth dorsal vertebræ. In the chest there was found nearly a pint of blood, chiefly in the cavity of the right pleura. There were several large spots of extravasated blood in the cellular tissue surrounding the pericardium. The lungs were filled with blood, and the bronchi had a dark red appearance upon their mucous surface.

In the abdomen there was a considerable quantity of blood effused under the peritoneum, between the bowels, and in the course of the mesentery. Blood also in great quantities between the muscles about the pelvis, but more particularly upon the right side. The hip-joint of the right side seemed to move with some freedom, unlike the opposite limb; and, when the muscles were cut through, there was a great deal of extravasated blood around the joint; the muscles that immediately surrounded it were much torn, as was also the capsule of the joint and the ligamentum teres. There was a fracture of the external rim of the acetabulum, and the head of the bone was a little displaced.

---

### *Fracture of the Fourth Cervical Vertebræ.*
### (St. Thomas's Hospital.)

Thomas Nunney, a porter, aged twenty-four, was admitted into Luke's Ward, at five P.M. Jan. 21st, 1829, under Mr. Tyrrell.

About half an hour previously, he had been carrying a weight of $1\frac{1}{2}$ cwt., when his foot slipping, he fell, and he believes that the load fell upon him. He was insensible for a short time, and when consciousness returned he found himself utterly incapable of using his hands or arms. When brought in, he was in a state of great depression. The pulse was below forty, and feeble; the surface was pale and cold; the breathing not affected.

When he had been placed in bed, a more careful examination was made. There was a complete loss of sensation of every part of the surface below an imaginary line drawn round the body, opposite to the sternal extremity of the fourth rib. It is not meant that feeling ceased here suddenly; for an inch or two below this point it gradually became less and less distinct, until quite extinguished.

There was very considerable loss of feeling in the arms. The numbness was greatest in the hands, less in the forearms, and still less in the shoulders. When handled at all roughly about the shoulders, he complained of a pricking sensation.

There was also an extensive deprivation of the power of motion. This was completely lost in all the voluntary muscles situated below the part already mentioned as that at which feeling ceased, viz. the fourth rib. Some ability to move the arms still remained, but it was restricted to slowly rotating the humerus to the extent of two or three inches.

Priapism, in an excessive degree, took place shortly after the injury.

The intellect did not appear entirely to have escaped injury. There was evidently slowness of comprehension, and hesitation in forming the proper answer. He also spoke very inarticulately; but it was difficult to determine how much of this effect might be attributed to the accident, as his friends said that he always stammered; and, from cold, his teeth chattered in a very remarkable degree. Although he complained much of cold, the surface was not below the natural standard of temperature. It was thought that he still retained the feeling of heat and cold in the parts dead to every other sensation; but of this there was some doubt, and unfortunately it was not put to the test of experiment.

A very careful examination of the spine was made by Mr.

Green, in the absence of Mr. Tyrrell. No fracture of any vertebræ could be felt, but there was tenderness over the fourth, fifth, and sixth cervical, and the patient complained of pain there when his head was moved. Mr. Green recommended that, in the event of complete reaction taking place, he should be bled to such an extent as the pulse might warrant.

Nine P.M.—Some reaction; pulse fifty-five, and stronger, but still weak; complains much of cold; some tympanitic swelling of the abdomen; no desire to evacuate the bladder or rectum. The urine was drawn off.

22d, ten A.M.—Pulse fifty, very feeble. More difficulty in speaking, and greater slowness of comprehension. Decidedly more feeling in the arms and more power of motion, as he can, with an effort, raise them from the bed, but they immediately fall again. No stool.—Ænema commune.

23d, ten A.M.—Pulse thirty-five, feeble: pupils contracted, but not equally, one more so than the other. Increased hesitation in speaking, and a tendency to delirium. The amendment which had taken place with regard to the arms has disappeared; and the priapism, incapability of emptying the bladder, obstinate constipation, and paralysis of the lower half of the body, continue unchanged. He complains of feeling very drowsy, but cannot sleep.

Ten P.M.—Pulse twenty; surface of the abdomen and thorax very cold, extremities less so. Intellect quite disordered, as, when asked a question, he begins to talk on a totally different subject; has been rambling all the evening. No stool.—Two enemata this day.

24th.—Has continued in the same state, talking almost continually, until four this morning, when he died, without having been insensible more than a few minutes.

There was no stool up to the time of his death. The urine had not begun to dribble away, nor had it become ammoniacal.

He did not sleep from the time of the accident, although often expressing a desire to do so. The breathing appeared easy from first to last. No unusual action of the sterno-mastoid or trapezii muscles was ever observed.

*Examination, forty-eight hours after death.*—The external surface of the body presented nothing remarkable. No displacement of the spinous process of any vertebra could be felt. The integuments and muscles were dissected from the whole length of the spinal column, and a fracture was then found running through the right side of the arch of the fourth cervical vertebra, very near the articular process. Under this a small clot of blood was found lying upon the theca, or extension of dura mater covering the cord. This covering was entire, and did not present any marks of inflammation. From the nature of the fracture, there was necessarily no displacement of the arch of the vertebra, and therefore no compression of the cord. The theca was next laid open: it contained more serous fluid than usual. Opposite to the fracture the cord appeared swollen, as if from blood extravasated within it. Its remaining membranes were there more injected than usual. A section was afterwards made through this portion of the cord, as it was found that blood really was extravasated through its substance, appearing as innumerable red points, intimately mingled with the natural colour of the medulla. When the cord was removed, another fracture was detected, extending longitudinally through the body of the fourth cervical vertebra.

The brain was next examined. The vessels of the pia mater were unusually full, both veins and arteries. On the surface of the cerebellum, under the pia mater, there was a considerable quantity of blood extravasated, in very thin layers or streaks. The substance, both of cerebrum and cerebellum, was healthy.

On the fore part of the spinal column, opposite to the fracture, there was much extravasated blood. Thinking, from the great depression of the heart's action, that the superficial cardiac nerves might be compressed, Mr. Tyrrell dissected down to their origin, and found that they were involved in coagulated blood.

The lungs were filled with dark blood, as were the right chambers of the heart. The colon and rectum were filled with hard feces. The bladder and every other viscus were healthy.

